# Predicting Norovirus in England Using Existing and Emerging Syndromic Data: Infodemiology Study

**DOI:** 10.2196/37540

**Published:** 2023-05-08

**Authors:** Nikola Ondrikova, John P Harris, Amy Douglas, Helen E Hughes, Miren Iturriza-Gomara, Roberto Vivancos, Alex J Elliot, Nigel A Cunliffe, Helen E Clough

**Affiliations:** 1 Institute of Infection, Ecological and Veterinary Sciences University of Liverpool Liverpool United Kingdom; 2 National Institute for Health and Care Research Health Protection Research Unit in Gastrointestinal Infections University of Liverpool Liverpool United Kingdom; 3 Institute for Risk and Uncertainty University of Liverpool Liverpool United Kingdom; 4 Field Service, Health Protection Operations United Kingdom Health Security Agency Liverpool United Kingdom; 5 Gastrointestinal Infections and Food Safety (One Health) Division United Kingdom Health Security Agency London United Kingdom; 6 Real-time Syndromic Surveillance Team Health Protection Operations United Kingdom Health Security Agency Birmingham United Kingdom; 7 Centre for Vaccine Innovation and Access PATH Geneva Switzerland; 8 National Institute for Health and Care Research Health Protection Research Unit in Emerging and Zoonotic Infections University of Liverpool Liverpool United Kingdom

**Keywords:** syndromic data, syndromic surveillance, surveillance, infodemiology, norovirus, Google Trends, Wikipedia, prediction, variable importance, mental model, infoveillance, trend, gastroenteritis, gastroenterology, gastroenterologist, internal medicine, viral disease, viral, virus, communicable disease, infection prevention, infection control, infectious disease, viral infection, disease spread, big data, Granger causality framework, predict, model, web-based data, internet data, transmission

## Abstract

**Background:**

Norovirus is associated with approximately 18% of the global burden of gastroenteritis and affects all age groups. There is currently no licensed vaccine or available antiviral treatment. However, well-designed early warning systems and forecasting can guide nonpharmaceutical approaches to norovirus infection prevention and control.

**Objective:**

This study evaluates the predictive power of existing syndromic surveillance data and emerging data sources, such as internet searches and Wikipedia page views, to predict norovirus activity across a range of age groups across England.

**Methods:**

We used existing syndromic surveillance and emerging syndromic data to predict laboratory data indicating norovirus activity. Two methods are used to evaluate the predictive potential of syndromic variables. First, the Granger causality framework was used to assess whether individual variables precede changes in norovirus laboratory reports in a given region or an age group. Then, we used random forest modeling to estimate the importance of each variable in the context of others with two methods: (1) change in the mean square error and (2) node purity. Finally, these results were combined into a visualization indicating the most influential predictors for norovirus laboratory reports in a specific age group and region.

**Results:**

Our results suggest that syndromic surveillance data include valuable predictors for norovirus laboratory reports in England. However, Wikipedia page views are less likely to provide prediction improvements on top of Google Trends and Existing Syndromic Data. Predictors displayed varying relevance across age groups and regions. For example, the random forest modeling based on selected existing and emerging syndromic variables explained 60% variance in the ≥65 years age group, 42% in the East of England, but only 13% in the South West region. Emerging data sets highlighted relative search volumes, including “flu symptoms,” “norovirus in pregnancy,” and norovirus activity in specific years, such as “norovirus 2016.” Symptoms of vomiting and gastroenteritis in multiple age groups were identified as important predictors within existing data sources.

**Conclusions:**

Existing and emerging data sources can help predict norovirus activity in England in some age groups and geographic regions, particularly, predictors concerning vomiting, gastroenteritis, and norovirus in the vulnerable populations and historical terms such as stomach flu. However, syndromic predictors were less relevant in some age groups and regions likely due to contrasting public health practices between regions and health information–seeking behavior between age groups. Additionally, predictors relevant to one norovirus season may not contribute to other seasons. Data biases, such as low spatial granularity in Google Trends and especially in Wikipedia data, also play a role in the results. Moreover, internet searches can provide insight into mental models, that is, an individual’s conceptual understanding of norovirus infection and transmission, which could be used in public health communication strategies.

## Introduction

Norovirus is the most prevalent agent causing intestinal infection, associated with approximately 18% of cases of acute gastroenteritis worldwide [1]. This highly contagious pathogen is mostly transmitted from person-to-person [2] but can also spread via contaminated surfaces [3,4]. Outbreaks of norovirus commonly occur in enclosed settings and can be particularly disruptive in health care context, such as hospitals and care homes [5]. A previous study suggested that norovirus causes between 3.3 and 4.1 million infections in England annually [6]. There are no licensed vaccines [7] or specific treatment available for norovirus infection [8].

Norovirus surveillance in England is conducted by the UK Health Security Agency (UKHSA) and consists of routine laboratory reporting of confirmed cases, suspected or confirmed outbreak reporting, real-time syndromic surveillance, and molecular surveillance of circulating strains. Laboratory-confirmed norovirus infections are reported to the Second-Generation Surveillance System (SGSS), which routinely collects information on infectious diseases in England. Norovirus is not a notifiable causative agent under the UK Health Protection (notification) Regulations (2010) [9]; notwithstanding, it is recommended that laboratories report it voluntarily [10]. This regulation is one of the reasons for variation in levels of norovirus reporting across regions [11]. A prospective study conducted in 2009/2010 [12] demonstrated that norovirus is widely underreported in the community.

Detection of increasing disease levels in the community by syndromic surveillance is generally faster than traditional surveillance approaches, though may not be pathogen-specific, as it relies on symptoms rather than confirmed diagnosis [13]. In England, syndromic surveillance makes use of multiple health data streams including National Health Service (NHS) 111, a telephone and web-based health service whereby the public can obtain advice on health issues via trained health advisors [14]. Additionally, data on general practice (GP) physician in-hours and out-of-hours consultations are collected, among others. These existing syndromic data sources [15] were previously used in studies focused on defining early warning indicators and algorithms for gastrointestinal illness [16] and norovirus [17] and also real-time predictions informing forecasts of hospital admissions due to respiratory illness [18].

In addition to existing syndromic surveillance systems, multiple emerging syndromic data sources have been identified with the potential to be used for public health benefits. One of the most popular among researchers is relative search volumes (RSVs) provided by Google Trends [19]. A systematic review [20] suggested that many surveillance-focused studies using RSV found moderate to strong correlations with the studied disease. Further, Wikipedia, another emerging data source, was used successfully to predict influenza-like illness in the United States [21] and globally [9], even though the geographical relevance is based only on the language spoken in the country. Such studies demonstrated that emerging data could be used to predict increase in illness in the community ahead of other pre-existing surveillance systems and indicators.

The focus of this study is to assess the value of existing and emerging syndromic data sources in the prediction of confirmed laboratory reports of norovirus infection captured by national surveillance. Laboratory confirmation of cases contains a built-in delay from sample collection to final reporting to the computerized system, whereas existing syndromic data in England are available on a near real-time, daily basis. A short-term forecasting model that can provide timely predictions of the number of norovirus reports would be helpful in planning and coping with winter pressures and mitigating the impacts of norovirus on the health service.

## Methods

### Data

#### Laboratory Data

Data were acquired from the UKHSA SGSS, which routinely collects information on infectious diseases in England. Weekly counts of norovirus laboratory reports were used as a proxy for norovirus activity. Norovirus reports were aggregated for each of 6 age groups—0-4, 5-14, 15-24, 25-44, 45-64, and ≥65 years and each of 9 regions of England representing UKHSA geographical areas. This grouping provides 15 dependent variables in total, addressed by individual models.

#### Existing Syndromic Data

We obtained weekly counts of GP in-hours consultations for diarrhea, vomiting, and gastroenteritis, and NHS 111 calls for diarrhea and vomiting. GP in-hours consultations and NHS 111 calls are existing syndromic data sources and were provided in an aggregated format for each region and age group. Regional data were matched to the regions of norovirus laboratory reports, while age groups were considered as separate variables, for example, NHS 111 calls of those aged 25-44 years; this resulted in 30 variables per model (see Multimedia Appendix 1).

#### Emerging Syndromic Data

Regarding emerging data sets, RSVs extracted from Google Trends and English Wikipedia page views were analyzed. Google Trends data were accessed via an open-source application programming interface (API) implemented in R called “gtrendsR” [22]. Google Trends provides England-specific aggregation, but the weekly format begins each Sunday, which is not in line with the international standard (ISO 8601). Besides region, search volumes can be extracted based on a specific category such as “Health” [23]. However, this was not specified to avoid decreasing the variance of the individual time series, as searches for norovirus and related topics are already low relative to all searches. Further details about our search term selection process are described below.

Page views were extracted via public API [24] from English Wikipedia as data are unavailable regionally. Daily page views were aggregated to weeks using *zoo* package of R software (R Foundation for Statistical Computing) [25]. We identified 6 relevant Wikipedia pages—“Gastric flu,” “Stomach flu,” “Norovirus,” “Gastroenteritis,” “Vomiting,” and “Diarrhoea.”

#### Internet Search Terms Selection

To avoid introducing bias into the search term selection and maximize the discovery of relevant search terms, the following process was followed: (1) keywords for the initial search were identified; (2) correlated search terms for every keyword within the last 5 years were automatically downloaded with an R script (see Multimedia Appendix 2); (3) the current selection of search terms was manually reviewed to see whether any known synonyms of norovirus are missing— if yes, another keyword was introduced; (4) all the search terms were lagged by 1 week and combined with norovirus reports for each age group and region separately; and (5) correlation coefficients were calculated, and search terms with coefficients under 0.20 signifying a weak relationship with the outcome variables were removed.

We began with the terms “norovirus” and “gastroenteritis,” and, following review, we added “stomach flu” and “vomiting.” This yielded 127 unique search terms in total (see Multimedia Appendix 1).

#### Time Period and Data Preparation

Since Wikipedia page views for the topics of interest became available via API in July 2015, we restricted other data sets to this starting point. Furthermore, considering the impact of the COVID-19 pandemic on the laboratory data in the years 2020 and 2021 [26,27], we excluded the pandemic period by curtailing the data analysis to 2019.

Combining the existing and emerging syndromic data resulted in 163 variables (see Multimedia Appendix 1). To further reduce irrelevant signals, we removed variables with a weak correlation coefficient (*r*<0.20) for each norovirus group (the syndromic data were lagged by 1 week). In the case of the 5-14 years age group, this resulted only in 2 syndromic variables. For the rest of the groups, the number of preselected variables ranged from 25 to 65.

### Ethical Considerations

As only nonidentifiable data were used in this study, the University of Liverpool Research Integrity and Ethic committee confirmed that ethical approval was not required (Reference: 7489).

### Statistical Analysis

#### Overall Approach

Due to biases that can be introduced by a particular method, we used 2 approaches to understand the predictive power of existing and emerging syndromic data sources. The first approach was based on the concept of Granger causality, emphasizing the importance of flow of time, that is, cause precedes effect. The second approach used random forest modeling, a machine learning algorithm widely used for variable selection in the field of data science. We used R [28] and RStudio [29] to perform the analysis.

#### Granger Causality

The time series data were assessed for stationarity (ie, seasonality and trend) as this is assumed by Granger causality [30]. After seasonality and trend were removed, all variables required differencing, that is, a transformation of the original time series to differences between consecutive values. The augmented Dickey-Fuller test [31] was used to test stationarity. We then fitted a vector autoregressive (VAR) model to every combination of group-relevant syndromic variables and norovirus reports for each age group and region separately. For example, norovirus reports in London and “Gastroenteritis” Wikipedia page views would create one combination. Both variables in the combination were lagged, that is, delayed, by 5 weeks giving us 5 versions of the same variable from consecutive time points. We used VAR implementation from *vars* package of R software [32], which also includes a function to check for model stability. For more detailed description of VAR models and Granger causality in the human health context, see Bose et al [33].

The Wald test was then used to determine whether syndromic data in the given category precede norovirus reports. It was also possible to identify the opposite relationship, that is, norovirus activity precedes syndromic data. Granger causality framework refers to predictive relationships as “Granger-causing,” so syndromic data can Granger-cause norovirus activity, conversely norovirus activity can Granger-cause changes in syndromic data.

*P* values were corrected with the optimized false discovery rate [34] due to multiple hypothesis testing. The target level of significance was 5%.

#### Random Forest Modeling

Unlike the Granger causality framework, random forest modeling does not require stationarity, and so variables were not differenced. However, seasonality was removed, and data normalized to range from 0 to 1. The normalization step is important in random forests since the concrete variable values can impact the variable ranking and performance [35]. Additionally, syndromic variables were lagged by 1 and 2 weeks. We then fitted a random forest model for every age and regional category separately, which gave 15 models with norovirus laboratory reports as the outcome, that is, dependent variable and all group-relevant existing and emerging syndromic variables as predictors, that is, independent variables. Normally, distributed random noise was used to indicate which syndromic variables add value; that is, syndromic variables had to achieve better variable importance score than random noise. Explained variance, root mean square error on the out-of-bag data, and variable importance are reported based on 1000 trees. The out-of-bag data refer to the data points reserved for model validation only, and out-of-bag error is an estimate for predictive performance. Explained variance and root mean square error averaged over the 15 models determined whether to lag the syndromic data by 1 or 2 weeks.

Two metrics were used to assess variable importance: (1) decrease in the mean square error and (2) node purity. Mean square error decrease is based on permutation of syndromic variables, one at a time, in the out-of-bag section of the data. The average decrease across the individual trees is reported. Node purity is a tree-specific variable importance metric. In the regression context, node purity indicates the total decrease in node impurities from splitting on the variable measured by residual sum of squares averaged over all trees. In other words, node purity is a measure of how well a given variable decreases variance in the outcome. We used random forest implementation from the *rf* package of R software [35] to fit the models and assess variable importance.

#### Combining Approaches

The syndromic variables preceding norovirus laboratory reports are considered influential predictors in the Granger causality framework. The precedence is based on a significance test. On the other hand, according to random forest modeling, influential predictors are those syndromic variables that achieve higher variable importance than the random noise, that is, no significance test is performed.

The 2 random forest metrics are normalized to a 0-1 scale where values close to 1 represent high variable importance for a particular model. Only the top 10 variables in each method are displayed, allowing for simpler interpretation. This provides between 10 and 30 predictors per group or region. If a variable was influential according to Granger causality framework, but not in the random forest for the given age group or a region, it is assigned a negative value (−1) for random forest metrics. The R code using *ggplot2* [36], *dplyr* [37], and *stringr* [38] packages to produce the figures is available (see Multimedia Appendix 3).

## Results

### Granger Causality

Based on the Granger causality framework, majority of the group-relevant syndromic variables were assigned to the “not predicting” category, that is, there was no significant relationship between the independent and the dependent variable in time. Our analysis highlighted 11 unique predictors across existing (4) and emerging (7) syndromic data that preceded changes in norovirus activity; 2 predictors were significant both ways, in that they predicted norovirus activity but norovirus activity also predicted them (Table 1).

Additionally, 18 variables were Granger-caused by norovirus activity including internet searches for “flu symptoms,” “how long does norovirus last,” “norovirus incubation,” “symptoms norovirus,” “vomiting bug,” Wikipedia page for diarrhea, in-hour GP visits for gastroenteritis in those aged 25-44 and ≥65 years, and NHS 111 calls due to vomiting in those aged 15-24 years.

Different predictors were significant for different age groups and regions. For example, GP visits due to vomiting in small children aged 0-4 years preceded norovirus activity only in the 45-64 years age group model. However, the views of the *Gastroenteritis* Wikipedia page preceded norovirus reports in the East of England, South East, and ≥65 years age group. The Granger-causing was significant both ways in the South East model.

**Table 1:** Wald test: the most influential variables predicting norovirus laboratory reports in respective age- and region-specific models.

Syndromic data		Norovirus activity	*P* value
**Variables Granger-causing norovirus laboratory reports**			
	NHS^a^ 111 calls due to vomiting among individuals aged 5-14 years	Laboratory reports among individuals aged 25-44 years	.002
	NHS 111 calls due to vomiting among individuals aged 0-4 years	Laboratory reports among individuals aged 0-4 years	.001
	GP^b^ visits due to vomiting among individuals aged 0-4 years	Laboratory reports among individuals aged 45-64 years	.001
	GP visits due to vomiting among individuals aged ≥65 years	Laboratory reports from the West Midlands	.001
	Views of Wikipedia’s *Gastroenteritis* page	Laboratory reports among individuals aged ≥65 years	<.001
	Views of Wikipedia’s *Gastroenteritis* page	Laboratory reports from the East of England	<.001
	Search term “norovirus contagious”	Laboratory reports among individuals aged 25-44 years	.002
	Search term “norovirus first symptoms”	Laboratory reports from the East of England	.001
	Search term “norovirus”	Laboratory reports from the East of England	.002
	Search term “norovirus incubation period”	Laboratory reports from London	.001
	Search term “stomach bug”	Laboratory reports among individuals aged 0-4 years	.002
	Search term “stomach pain”	Laboratory reports from the North East	<.001
**Granger-causing significant both ways**			
	NHS 111 calls due to vomiting among individuals aged 25-44 years	Laboratory reports among individuals aged 0-4 years	.002
	Views of Wikipedia’s *Gastroenteritis* page	Laboratory reports from the South East	<.001

^a^NHS: National Health Service.

^b^GP: general practice.

### Random Forest

The predictive relationship in this section was investigated only one way—whether existing and emerging syndromic data predict norovirus laboratory reports. The random forest models with syndromic data lagged by 1 and 2 weeks were mostly similar, except North East, London, and South East (Table 2). The North East model’s variance explained increases from ~8% to ~20% in random forest with lag of 2 weeks. The highest percentage of variance explained was in the 65+ years group (~60%, lag 2), 0-4 years group (~49%, lag 1), London (~47%, lag 1), and East of England (~44%, lag 1). However, errors were slightly lower in the random forest with lag 2.

**Figure 1:**
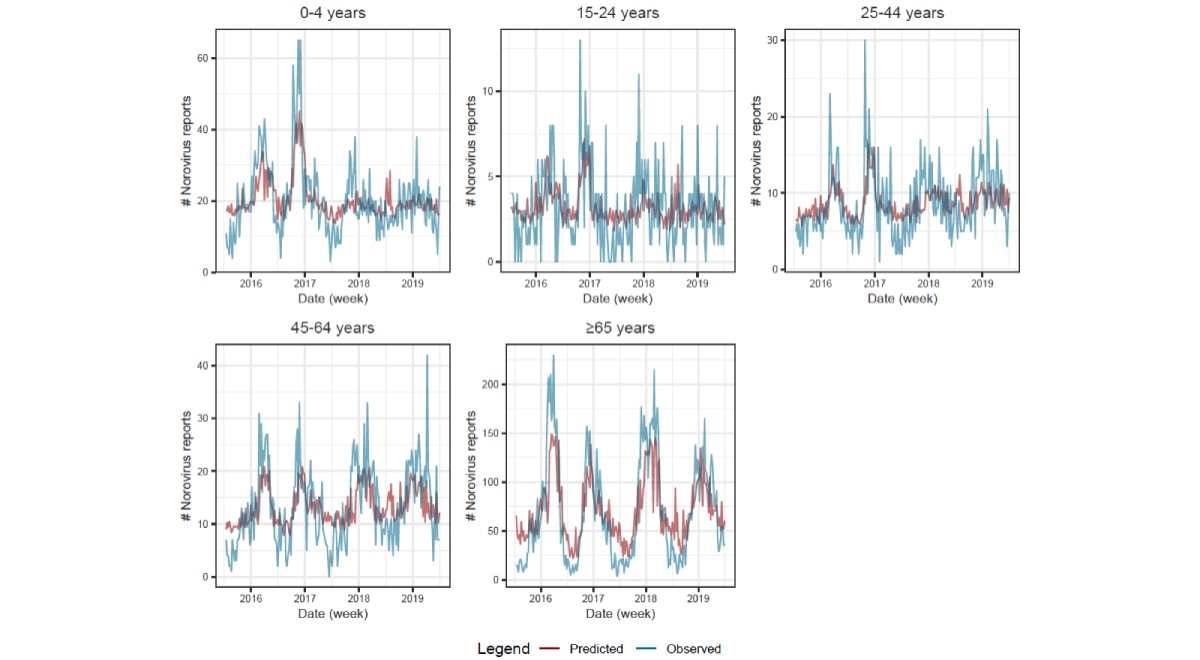
Random forest fit for each age group (lag=2; no predictors were identified for the 5-14 years age group).

**Figure 2:**
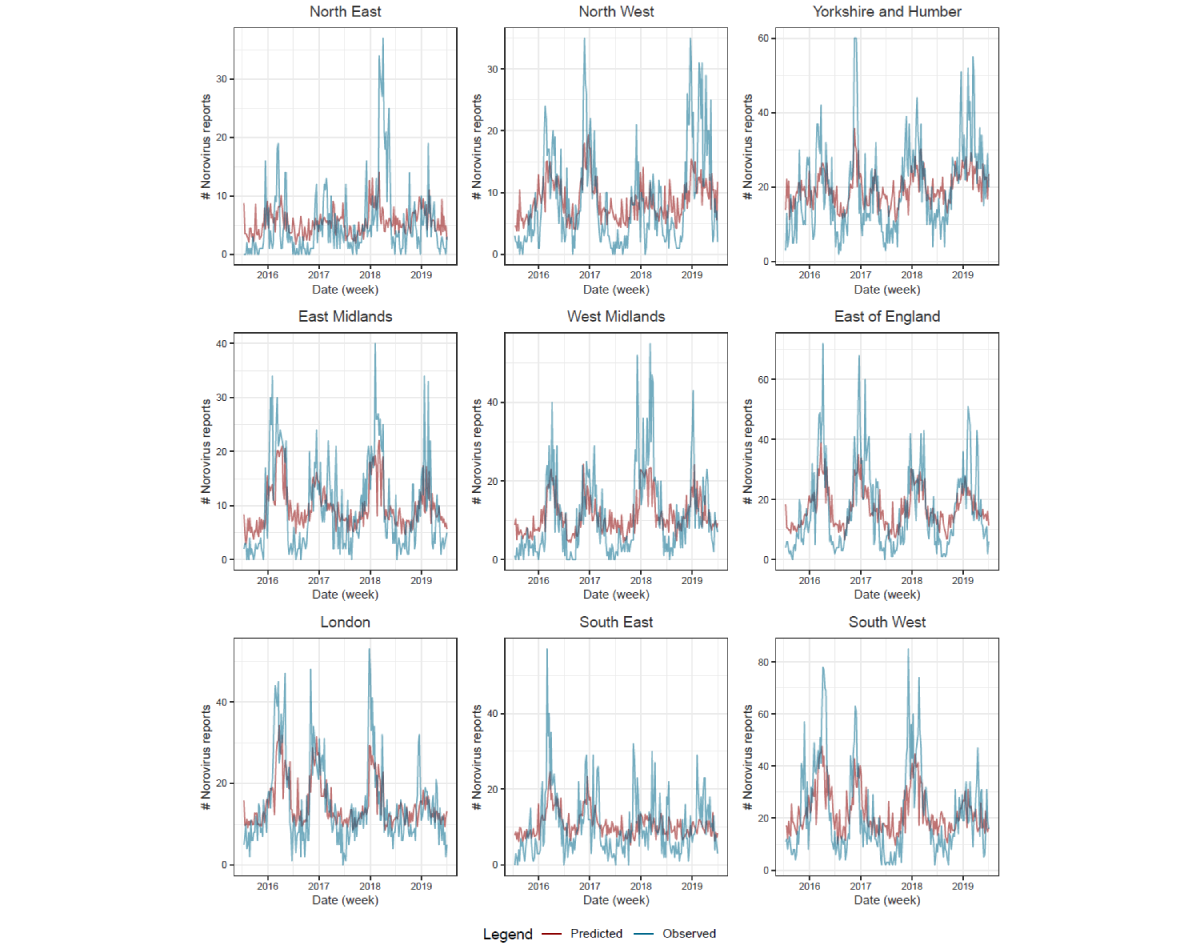
Random forest fit for each region (lag=2).

Fitted values are presented for 5 age groups (Figure 1) and 9 regions (Figure 2). We were not able to fit a random forest model to predict norovirus laboratory reports in 5-14 years age group with the syndromic predictors. This was due to a low number of correlated syndromic variables and low variance of the laboratory reports in this age group, with many weeks reporting zero.

Similar to the Granger causality results, different predictors were important in the individual models. For example, vomiting-related NHS 111 calls in those aged 5-14 years were important in all age groups and 4 regional models (North West, Yorkshire and Humber, West Midlands, and East of England), while the GP in-hour visits due to vomiting in 15-24 years were an important variable only in the model predicting laboratory reports in 15-24 years age group. The internet searches for “flu symptoms” occurred in almost all of the 15 laboratory reports–predicting models as an important predictor except 0-4 years age group where the syndromic data were lagged by 2 weeks. There was also a pattern of “flu symptoms” appearing more important in older age groups (45+ years) compared to younger groups.

**Table 2:** Random forest (RF) performance comparison: predicting norovirus laboratory reports in specific age groups and regions—existing and emerging syndromic variables were lagged by 1 or 2 weeks.

Predicting laboratory reports for each group and region			RF (lag=1)					RF (lag=2)		
			Explained %		RMSE^a^ (bootstrap)		Explained %			RMSE (bootstrap)
**Age group (years)**										
	0-4	48.8		8		43			8.2	
	5-14	—^b^		—		—			—	
	15-24	13.14		2.2		6			2.3	
	25-44	25.18		4		20			4.3	
	45-64	32.16		6.6		33			6.5	
	≥65	55.69		40.5		60			36.6	
**Region**										
	North East	7.50		6.6		19.91			6.2	
	North West	30.39		7.2		34.06			6.5	
	Yorkshire and Humber	29.79		11.3		26.41			10.9	
	East Midlands	39.42		6.9		42.19			7.1	
	West Midlands	35.11		9.73		32.44			9.6	
	East of England	44.46		12		42			11.6	
	London	47.1		8.7		35.45			8.5	
	South East	18.1		8.8		12.6			8.3	
	South West	35		15.7		38.57			15.1	

^a^RMSE: root-mean-square error.

^b^Not available.

### Combined Results

**Figure 3:**
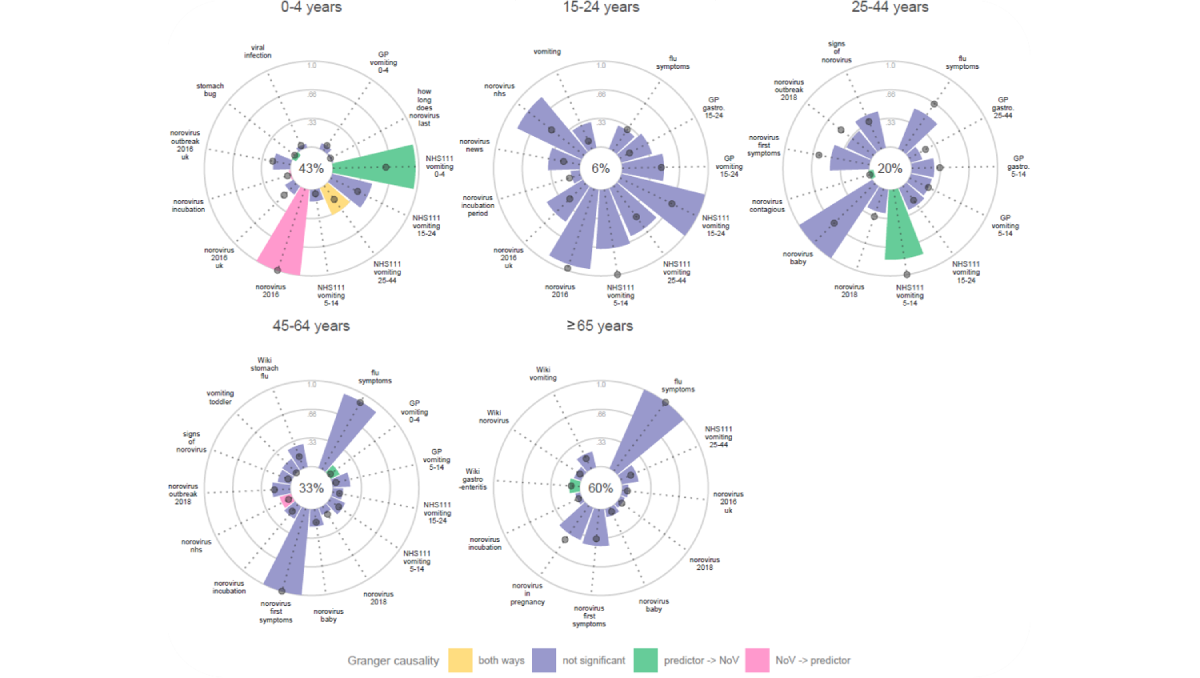
The most influential variables predicting norovirus (NoV) laboratory reports in each age group. (The height of the bar indicates variable importance based on node purity, and the position of the dark gray dot indicates mean square error. Variance explained by the random forest model is shown as a percent value in the middle. Significance as judged by q value based on the Granger causality framework is marked in green and pink or yellow if detected both ways. Only the top 10 variables per method are displayed, that is, between 10 and 30 predictors per group. When a variable is not important based on random forest metrics, the bar points inward and the dot is at 0 position. Existing syndromic data sources are marked based on the source, that is, NHS 111 and GP. Wikipedia pages are marked with “Wiki” and the rest of selected variables are relative search volumes from Google Trends.) GP: general practice; NHS: National Health Service.

Both methods indicated distinct syndromic predictors in specific laboratory-reporting models with some overlap. For example, the methods are conflicting in the 0-4 years age group (Figure 3), where internet searches for “stomach bug” Granger-caused norovirus laboratory reports but the variable was ranked as unimportant by random forest modeling. On the other hand, the most important predictor identified by random forest (internet searches for norovirus 2016) is a variable that was preceded by norovirus laboratory reports, that is, changes in the variable occurred after changes in the norovirus activity. However, both random forest and Granger-causality framework identified, and NHS 111 vomiting calls concerning young children (0-4) as an influential variable.

Norovirus activity in adults aged 25-44 years was predicted by NHS 111 calls on behalf of school children (5-14 years) in both methods, while internet searches for “norovirus contagious” was identified only by Granger causality.

**Figure 4:**
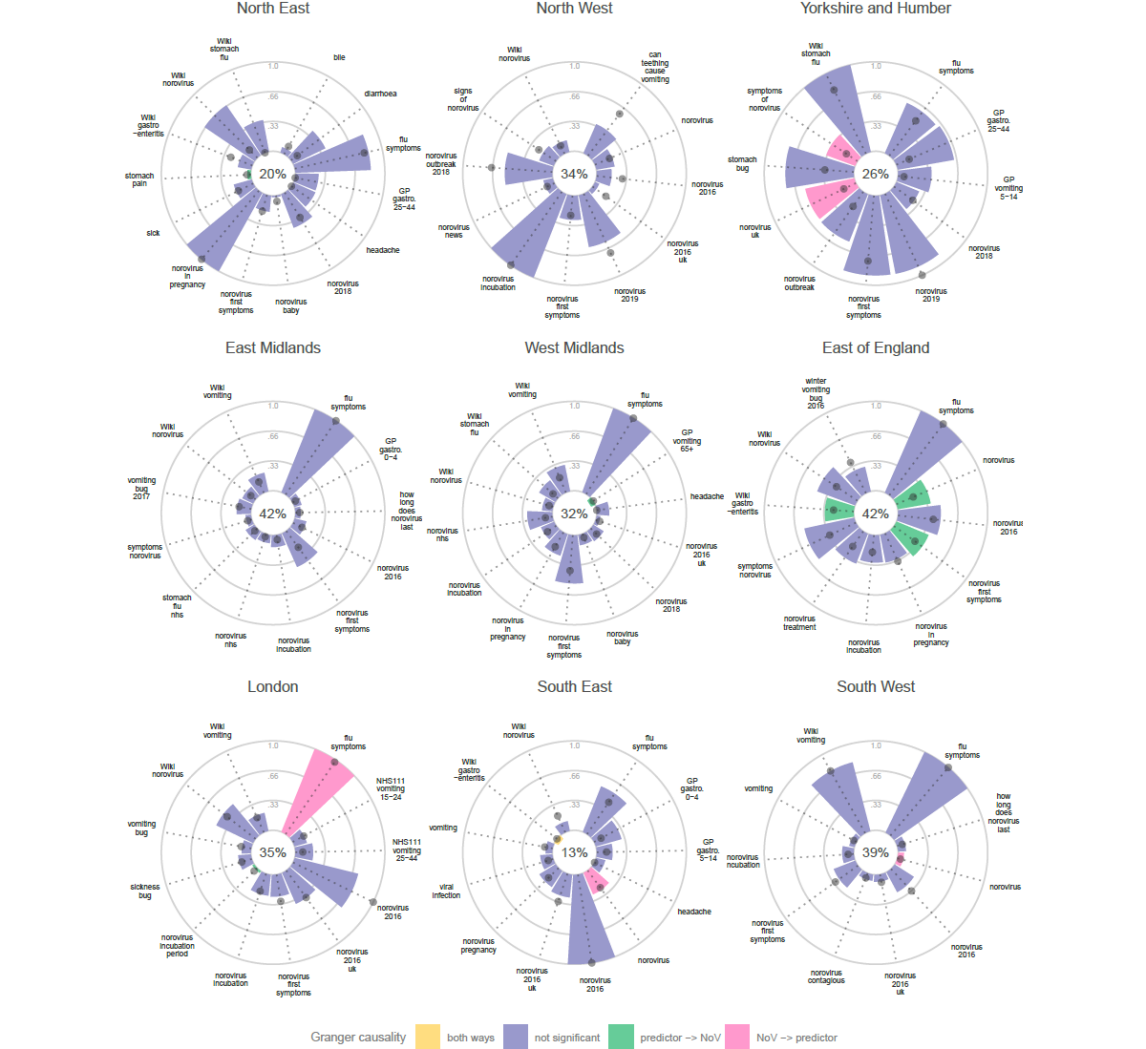
The most influential variables predicting norovirus (NoV) laboratory reports in each region. (The height of the bar indicates variable importance based on node purity, and the position of the dark gray dot indicates mean square error. Variance explained by the random forest model is shown as a percent value in the middle. Significance as judged by q value based on Granger causality framework is marked in green and pink or yellow if detected both ways. Only top 10 variables per method are displayed, that is, between 10 and 30 predictors per group. When a variable is not important based on random forest metrics, the bar points inward and the dot is at 0 position. Existing syndromic data sources are marked based on the source, that is, NHS 111 and GP. Wikipedia pages are marked with “Wiki” and the rest of selected variables are relative search volumes from Google Trends.) GP: general practice; NHS: National Health Service.

Norovirus activity in the East of England was explained well with the random forest model—58 variables selected as important including the ones from the Granger causality framework (Figure 4). Internet search volumes for the term “flu symptoms” was highlighted by both measures in the random forest approach in 5 regions—West and East Midlands, East of England, London, and South West, but the Granger causality approach highlighted none of them as an influential predictor of norovirus laboratory reports. The “flu symptoms” term was significant only as being preceded by norovirus reports in London. In contrast, the selected influential variables from both methods seem to be more correlated in the East of England.

## Discussion

### Principal Results

Our findings suggest that both existing and emerging syndromic surveillance data can predict the number of laboratory reports of norovirus, but the success and specific variables vary across age groups and regions of England. In other words, syndromic data are less relevant for some age groups and regions than others. For example, the combined existing and emerging syndromic variables explained 60% variance in the ≥65 years age group model, 42% in the East of England, and only 13% in the South West. The variation can be due to contrasting public health reporting, diagnostic testing practices, and resource constraints between regions. Data biases in the laboratory and syndromic surveillance data could have also led to a poor predictive performance. For example, we could not find any influential predictors for the laboratory reports in the 5-14 years age group, which could be associated with the fact that the signal in these data is weak and requires different statistical approaches than those used here. Another factor is the population structure of specific regions since health information–seeking behavior differs across age groups [39].

The most influential predictors from emerging data sets included search terms such as “norovirus contagious,” “norovirus first symptoms,” “norovirus,” “norovirus incubation period,” “stomach bug,” “stomach pain,” “norovirus in pregnancy,” “norovirus baby,” “flu symptoms” and norovirus activity in specific years, for example, “norovirus 2016.” The year-specific search terms suggest instability of the relationship between some of these variables and norovirus activity from year to year. In other words, predictors relevant in one norovirus season may not substantially contribute in other seasons. This could be linked to the stochastic nature of laboratory-confirmed numbers of norovirus infections that are known to vary from season to season. Additionally, the fluctuations in search term relevance could be related to how and when the media cover norovirus outbreaks [40].

Our analysis suggests that Wikipedia page views are less likely to provide prediction improvements on top of Google Trends and Existing Syndromic Data. The page views for “Gastroenteritis” were predictive of norovirus laboratory reports in East of England and the 65+ years age group only, and other pages were on the lower end of the random forest variable importance. One reason for the lower importance of Wikipedia page views in the random forest is that the time series is correlated with other variables that already provided similar signals, so Wikipedia page views are ranked lower [35]. Also, the page views data are only available based on language, so the signal from other English-speaking countries brings unnecessary noise, that is, false signal.

Additionally, the Granger causality framework and random forest modeling results support the greater emphasis placed upon vomiting as a predictor of norovirus activity compared with diarrhea. Previous studies identified vomiting-related syndromic surveillance as preceding norovirus activity. Specifically, under 5 vomiting calls in the UK [17] and vomiting calls in all age groups in Ontario, Canada [41]. Moreover, our analysis showed that vomiting in the 5-14 years age group precedes norovirus activity in the 25-44 and random forest indicated this variable was also influential in other age groups. School-aged children were previously identified as a good indicator of influenza activity [42], suggesting the importance of social contact patterns in this age group for person-to-person transmission. This provides further support to the notion of school-based surveillance of acute infectious diseases such as norovirus [43].

### Existing and Emerging Syndromic Data and Norovirus Surveillance

The main advantage of existing syndromic surveillance data is the availability of grouping by age and region. This level of granularity is crucial for a holistic view of norovirus activity. For example, existing syndromic surveillance can indicate increased gastrointestinal symptoms in the London region. If the number of suspected or confirmed norovirus outbreaks increases in the same region, this provides further evidence to inform risk management and health protection actions.

In contrast, emerging syndromic data sets investigated in this study are not presented at this level. Most internet search volumes extracted from Google Trends are available for England only due to low norovirus-related volumes compared to other topics. Further, the Google Trends system lacks transparency about how the total searches are sampled, and extracting more than 5 search terms is not straightforward. Despite these challenges, RSVs can provide a relevant predictive signal for specific age groups and regions and potentially overall. However, these data are not well-suited for the triangulation of norovirus activity per se, and statistical modeling is required to extract and clean the signal. For instance, a short-term forecasting model can predict the number of confirmed cases a few weeks ahead; this way, the limited signal in the data can be leveraged. Since laboratory reporting is subject to delays, such a model based on Google Trends and other data sets could provide a better estimate of the expected number of reports. The forecasts could then be used in public health surveillance of norovirus, as illustrated in the US influenza forecasting competition [44].

Additionally, we found that the approaches assigned diverse levels of importance to the syndromic variables across the age- and region-specific models. This suggests that potential early warning and forecasting systems should consider regularization methods [45] that assign low weight to less relevant variables rather than removes them. Alternatively, to decrease the number of variables, the principal component analysis could be used [46].

### Internet Searches and Mental Models of Disease

Mental models and representations are well-studied psychological concepts, yet they have been only recently discussed in the context of risk communication [47] and public health policy [48]. Briefly, mental model refers to the implicit or explicit understanding of how something works. This study highlights the unrestricted nature of internet searches, which could provide insight into mental models of norovirus and other infections. Such insights could be used to guide qualitative methodologies such as semistructured interviews when exploring informational needs of health advice seekers on the internet [49].

Our analysis indicated RSVs for “flu symptoms” among the best predictors of norovirus laboratory reports in multiple regions. In other words, some people experiencing norovirus infection could be searching for symptoms of flu or stomach flu, among others. This is in line with a recent study from the United States where the searches correlated with norovirus activity included “stomach flu” [50]. People’s misunderstanding of a disease is crucial because it guides their behavior while experiencing the disease. For example, norovirus can survive on contaminated surfaces [3] but by thinking they have flu, people can underestimate the importance of cleaning and the risk of food contamination by a symptomatic individual. Furthermore, there seems to be a pattern where the searches for “flu symptoms” were more important in those 45 years or older, particularly those 65 years or older. The flu-like perception of norovirus could be related to historic public health messaging, and the fact that norovirus is a relatively recently discovered pathogen [51].

### Strengths and Limitations

The main advantage of this study is that we use 2 approaches to assess predictability. With the first approach based on Granger causality and significance testing, we capture the predictive potential of individual syndromic variables. The second approach uses random forest regression as a popular prediction algorithm, and this helps assess the predictive potential in the context of other variables. Additionally, the analysis highlights how different methods can highlight different explanatory variables and provide robust evidence of predictability when the results of both approaches are aligned. Future research could explore predictability in higher moments via the quantile dependence method [52] or test the predictive performance in a real-time norovirus prediction context.

Furthermore, we follow an automated correlation–based search term selection process for Google Trends. This helps avoid incorporating biases and assumptions about how the public might think about norovirus infections and what words they would use to describe symptoms. Automated selection has been shown to achieve great predictive performance in previous flu forecasting research [53]. Our approach also involved manual inspection to ensure that the search terms were relevant. It may have missed some norovirus-related search terms, but the goal of this analysis is to assess whether Google Trends can offer useful signals for norovirus prediction, not to provide an exhaustive list of norovirus search terms in England. Additionally, it is essential to note that the Google Trends tool [19] provides RSVs based on a sample of searches, not the total number, which can vary with each request.

The main limitations of our analysis are in the data. Norovirus infections or outbreaks often go unreported, leading to underreporting in national surveillance systems, so confirmed laboratory reports do not represent actual norovirus activity [12]. Other sources of syndromic surveillance data, such as health-based systems and web-based sources like Twitter, were not considered and could add additional predictive signals. Previous research also used data from a UK infection portal that captures health professionals’ web-based behavior [40], and such data could provide other signals in a predictive modeling context.

### Conclusions

In conclusion, our study aimed to assess the predictive potential of existing and emerging syndromic data for norovirus prediction, focusing on individual time series assessment and combined assessment of all variables in random forest modeling. The results show that existing and emerging data sources can help predict norovirus activity in England in some age groups and geographic regions, particularly with predictors concerning vomiting, gastroenteritis, and norovirus in vulnerable populations and historical terms such as stomach flu. However, syndromic predictors were less relevant in some age groups and regions, and those influential in one norovirus season may not contribute to another. The variation can be due to contrasting public health practices between regions and health information–seeking behavior between age groups.

In terms of public health surveillance, the critical advantage of existing syndromic surveillance is the high granularity in age and region. As a result, the data can be easily compared against other surveillance systems and immediately inform risk management. In contrast, emerging syndromic data are not well-suited for the triangulation of norovirus activity per se and require statistical modeling to extract and clean the signal. Our results emphasize the importance of automating the search terms selection process and provide insight into potentially successful predictive approaches. Specifically, we recommend using methods such as regularized regression that can handle high numbers of independent variables or extracting variance with principal component analysis.

Our findings have implications for public health surveillance and policy regarding norovirus and possibly beyond. The potential of mental models, such as those reflected in internet searches, in developing targeted and effective communication and knowledge mobilization strategies in the face of emerging situations is huge.

## Appendix

Multimedia Appendix 1 List of all variables.

Multimedia Appendix 2 R script to automatically download the relative search volumes.

Multimedia Appendix 3 R script code and data to produce the visualizations.

## Abbreviations

API: application programming interface
GP: general practice
NHS: National Health Service
RSV: relative search volume
SGSS: Second-Generation Surveillance System
UKHSA: UK Health Security Agency
VAR: vector autoregressive

## Data Availability

Routine surveillance data cannot be shared publicly because the provision of the data is dependent on the intended use. Raw norovirus data are available from UKHSA (EEDD@ukhsa.gov.uk). The R code generated during this study to find relevant Google Trends and the code and data to produce the final circular visualization are available as Multimedia Appendices 2 and 3, respectively. Public application programming interfaces to acquire Google Trends and Wikipedia are referenced [22,24].

## Disclaimer

NO is funded by Engineering and Physical Sciences Research Council (EPSRC) and Economic and Social Research Council (ESRC) Centre for Doctoral Training in Quantification and Management of Risk & Uncertainty in Complex Systems & Environments (grant EP/L015927/1). The funders had no role in study design, data collection and analysis, decision to publish, or preparation of the manuscript. NAC, RV, HEH, AJE, and NO are affiliated to and HEC is partially funded by the National Institute for Health and Care Research (NIHR) Health Protection Research Unit (HPRU) in Gastrointestinal Infections at the University of Liverpool, in partnership with the UK Health Security Agency, in collaboration with University of Warwick. AJE is affiliated to the NIHR HPRU in Emergency Preparedness and Response based at the University of East Anglia. NAC is a NIHR Senior Investigator (NIHR203756). The views expressed are those of the authors and not necessarily those of the NIHR, the Department of Health and Social Care, or the UK Health Security Agency.
