# Supplementary material for: Predicting Norovirus in England Using Existing and Emerging Syndromic Data: Infodemiology Study
Source: J Med Internet Res. 2023 May 8;25:e37540. doi: 10.2196/37540 (PMC10203923; doi:10.2196/37540)
Supplement: Multimedia Appendix 1 [file jmir_v25i1e37540_app1.pdf]

# Multimedia Appendix 1:
# Lists of variables

# Existing Syndromic Data: List of All Variables

**GP in-hours consultations** (24 variables):

- GP (in-hours) diarrhea 0-4 / 5-14 / 15-24 / 25-44 / 45-64 / 65+ ;
- GP (in-hours) vomiting 0-4 / 5-14 / 15-24 / 25-44 / 45-64 / 65+ ;
- GP (in-hours) gastroenteritis 0-4 / 5-14 / 15-24 / 25-44 / 45-64 / 65+

**NHS 111 telephone calls** (12 variables):

- NHS 111 diarrhea 0-4 / 5-14 / 15-24 / 25-44 / 45-64 / 65+ ;
- NHS 111 vomiting 0-4 / 5-14 / 15-24 / 25-44 / 45-64 / 65+

# Emerging Syndromic Data: List of All Variables

**Wikipedia Pageviews** (6 variables):

- English Language Wikipedia Pages - Gastric flu, Stomach flu, Norovirus, Gastroenteritis, Vomiting and Diarrhoea

**Relative Search Volumes from Google Trends** (127 variables):

- norovirus, symptoms norovirus, norovirus uk, diarrhea, norovirus diarrhea, norovirus nhs, symptoms of norovirus, norovirus incubation, norovirus treatment, norovirus outbreak, norovirus incubation period, norovirus 2016, stomach bug, norovirus spread, norovirus news, sickness bug, what is norovirus, gastroenteritis, norovirus contagious, rotavirus, food poisoning , norovirus 2018, how long does norovirus last, norovirus baby, norovirus vaccine, norovirus pregnancy, norovirus 2017, norovirus 2015, norovirus 2016 uk, how does norovirus spread, norovirus 2019, norovirus outbreak 2018, norovirus is not spread by, norovirus outbreak 2016 uk, can dogs get norovirus, norovirus first symptoms, how is the norovirus spread, how long does norovirus live, norovirus in pregnancy, can dogs catch norovirus, how to treat norovirus, norovirus in babies, what to eat after norovirus, how do you get norovirus, signs of norovirus, symptoms, viral gastroenteritis, gastroenteritis nhs, gastritis, gastroenteritis contagious, symptoms of gastroenteritis, gastroenteritis dogs, stomach pain, what is gastroenteritis, baby gastroenteritis, is gastroenteritis contagious, gastroenteritis in children, gastroenteritis in dogs, gastroenteritis treatment, gastroenteritis in babies, diarrhoea, dog gastroenteritis, gastroenteritis what to eat, how long does gastroenteritis last, bacterial gastroenteritis, stomach flu, hemorrhagic gastroenteritis, dioralyte, viral gastroenteritis nhs, gastroenterologist, signs of gastroenteritis, treatment for gastroenteritis, sickness and diarrhea, appendicitis, flu symptoms, stomach flu symptoms, stomach flu nhs, stomach cramps, gastric flu, what is stomach flu, how long does stomach flu last, stomach ulcer, vomiting, vomiting diarrhea, vomiting baby, vomiting and diarrhea, sick, dog vomiting, vomiting blood, vomiting bug, nausea, fever, vomiting bile, bile, vomiting nhs, projectile vomiting, vomiting toddler, cat vomiting, headache vomiting, headache, nausea and vomiting, diarrhoea and vomiting, vomiting after eating, children vomiting, vomiting and stomach pain, vomiting bug 2017, toddler vomiting no fever, vomiting and diarrhea at the same time, viral infection, pepto bismol, how to stop vomiting bile, winter vomiting bug 2016, winter vomiting virus 2015, cvs, can teething cause vomiting, child vomiting no fever, pancreatitis, best thing to eat after vomiting, ibs symptoms, vomiting emoji, vomiting and diarrhea in dogs, acid reflux vomiting, what to eat when sick, upset stomach, can tonsillitis cause vomiting, flu and vomiting, fever and vomiting
